# Compressive Volumetric Light-Field Excitation

**DOI:** 10.1038/s41598-017-13136-2

**Published:** 2017-10-25

**Authors:** David C. Schedl, Oliver Bimber

**Affiliations:** Faculty of Engineering and Natural Sciences, Johannes Kepler University, Linz, 4040 Austria

## Abstract

We explain how volumetric light-field excitation can be converted to a process that entirely avoids 3D reconstruction, deconvolution, and calibration of optical elements while taking scattering in the probe better into account. For spatially static probes, this is achieved by an efficient (one-time) light-transport sampling and light-field factorization. Individual probe particles (and arbitrary combinations thereof) can subsequently be excited in a dynamically controlled way while still supporting volumetric reconstruction of the entire probe in real-time based on a single light-field recording.

## Introduction

Volumetric recording of fluorescent activity is an essential tool in modern microscopy (e.g., for optogenetics^1–7^). Achieving fast readouts remains challenging, since the majority of microscopy techniques record volumes by scanning (e.g., confocal microscopy, light-sheet scanning^8^, and one- or two-photon SLM-based scanning techniques^9,10^).

Light-Field Microscopy (LFM)^7,11,12^, in contrast, captures the 4D incident light (e.g. by means of a microlens array (MLA) placed at the intermediate image plane of the imaging path), and thus supports the computation of a focal stack based on a single sensor recording. Optical phenomena in thick tissue such as scattering and optical aberrations prevent the application of traditional imaging techniques. Light-field recordings, however, encode these effects.

Pegard *et al*.^13^ applied LFM imaging to distinguish and localize 3D neuronal activity by non-negative matrix factorization^14^ during a training phase (a light-field video recorded at constant illumination during which the neurons fired randomly) to separate overlapping light-field signatures. Decomposition works as long as only few neurons fire simultaneously and at least once independently from other neurons in the training period (i.e., neural activity must be sparse in the temporal and spatial domains). Training generates a database of light-field signatures that allows fast readouts of neuronal activity while completely avoiding any image formation process.

We have explained in previous work^15^ how to concentrate light simultaneously at multiple selected volumetric positions by means of a 4D illumination light field that is computed directly from the appearance of the probe. We refer to this as *volumetric light-field excitation* (VLE), which may find applications in the field of optogenetics. In contrast to holographic projection with phase modulators^9,16,17^, light-field projection with spatial-light modulators (e.g. in combination with MLAs or realized with multiple interplaying SLMs^18,19^) does neither limit the excitation area or the illumination pattern^20^, nor suffers from spatially varying diffraction efficiency and the presence of zero-order diffraction spots, ghosting, and intensity fluctuations (speckles)^3,21^. With one sensor recording, we capture a 4D light field of the probe under full illumination and apply synthetic aperture rendering^22,23^ to compute a 3D focal stack or individual perspective images from it. The focal stack undergoes 3D deconvolution^24^ to produce a defocus-free z-stack. Within the z-stack, we select parts of the probe (3D-segmented fluorescent microspheres of typical neuron sizes of model organisms, such as C. elegans and zebrafish larvae) that are to be excited. From the selection in the z-stack, we then determine a 4D light-field mask that is projected simultaneously into the probe such that the light concentrated at the volumetric positions of the selection is maximized while the illumination of other regions is minimized. This approach, however, has several essential drawbacks: It requires precise 3D reconstruction of the probe from a focal stack. As deconvolution is ill-posed, this is not always possible and can lead to reconstruction errors. Furthermore, it requires precise calibration of all optical elements in the imaging and illumination paths of the microscope, and ignores scattering in the probe.

In this article, we explain how VLE can be converted to a process that entirely avoids 3D reconstruction, deconvolution, and calibration of optical elements while taking scattering in the probe better into account. For spatially static probes, this is achieved by an efficient (one-time) light-transport sampling and light-field factorization. Individual probe particles (and arbitrary combinations thereof) can subsequently be excited in a dynamically controlled way while still supporting volumetric reconstruction of the entire probe in real-time based on a single light-field recording.

## Compressive VLE

Equation 1 outlines the principle of *compressive volumetric light-field excitation* (CVLE):

1 $$[T][{\Phi }]=[Y]=[{\Psi }][X]$$

Let *Φ* be a sequence of illumination light fields (each column of the matrix represents one illumination light field), and *Y* be the corresponding sequence of imaging light fields (each column *y*_*i*_ represents the imaging light field recorded for the corresponding illumination light field in *ϕ*_*i*_), then the light-transport matrix *T* represents the entire transport of light from *ϕ* through the probe to *y* in the sense of *y* = *Tϕ*.

If *T* were known, then, for a given desired imaging light field *y*_*i*_, we could determine the corresponding illumination light field *ϕ*_*i*_ with *ϕ*_*i*_ = *T* ^−1^ *y*_*i*_ (or by solving *y*_*i*_ = *Tϕ*_*i*_ for *ϕ*_*i*_). Determining *T* by brute-force calibration (i.e., *Φ* is identity), however, is infeasible, as the number of measurements would have to match the resolution of the illumination light field (i.e. in the range of several thousands to more than one million).

Nevertheless, if we can assume that the emission of excited particles in the probe is isotropic, *T* contains a significant amount of redundancy. Compressive sensing principles^25^ can be applied to reduce the number of measurements to obtain a representation of *T* that suits our isotropy assumption.

If *Φ* is a sampling sequence, *Y* the corresponding imaging light fields and the probe isotropic, then there might exist a basis *Ψ* in which *Y* can be expressed with few coefficients *X* such that *Y* = *ΨX*. Note that matrices *Ψ* and *X* are much smaller than *Y* (i.e., contain fewer coefficients) and are thus compressed. The challenge in compressive sensing is to choose a proper sampling *Φ* such that *Y* is compressible. Furthermore, the number of samples in *Φ* can be (far) smaller than required by the Nyquist-Shannon sampling theorem (i.e., *Φ* is identity).

In fact, *Ψ* and *X* can be determined directly from *Y* by means of an efficient independent component analysis (ICA), as demonstrated by Pegard *et al*.^13^ for light-field microscopy imaging. This could be traditional non-negative matrix factorization^14^, one of its variants^13^, or a modern dictionary learning technique^26,27^. In contrast to the Pegard *et al*. approach^13^, however, our sampling is adaptively controllable by choosing the sequence of illumination light fields in *Φ*.

Once sparse representations of *T* and *Ψ* have been found, we can finally determine an illumination light field *ϕ*_*i*_ for a desired imaging light field *y*_*i*_, as explained above (i.e., with *ϕ*_*i*_ = *T* ^−1^ *y*_*i*_ or by solving *y*_*i*_ = *Tϕ*_*i*_ for *ϕ*_*i*_), composing the desired imaging light field *y*_*i*_ by a linear combination of the columns in *Ψ*.

A 3D reconstruction for the selection of the probe parts to be illuminated as in Schedl and Bimber’s^15^, which requires deconvolution and precise optical calibration, is not necessary. Furthermore, *Ψ* also contains the information about scattering in the probe, which can now be considered.

Note that *T* and *Ψ* have to be determined only once per probe as part of a training phase. Such training by recording a light-field video of the probe under constant illumination is also required in the approaches of Pegard *et al*.^13^ and Pnevmatikakis *et al*.^28^. After this training period, however, computation of volumetric light-field excitation patterns (i.e., solving for *ϕ*_*i*_) as well as 3D imaging and visualization of the excited probe by means of light-field imaging and synthetic aperture rendering^22,23^ or model-driven 3D estimations of probe particles^13,29^ can still be achieved in real time (i.e., at the speed of the camera exposure required).

## Light-Transport Sampling

Acquiring a robust estimate of the light-transport (*T*) more efficiently than with brute-force scanning is essential to computing accurate illumination light fields. We propose a parallel algorithm that acquires *T* for 4D light fields adaptively—similarly to the hierarchical 2D image sampling proposed by Sen *et al*.^30^. Furthermore, we reduce the number of recordings by applying an isotropy hypothesis that determines whether finer illumination levels are needed.

**Figure 1:**
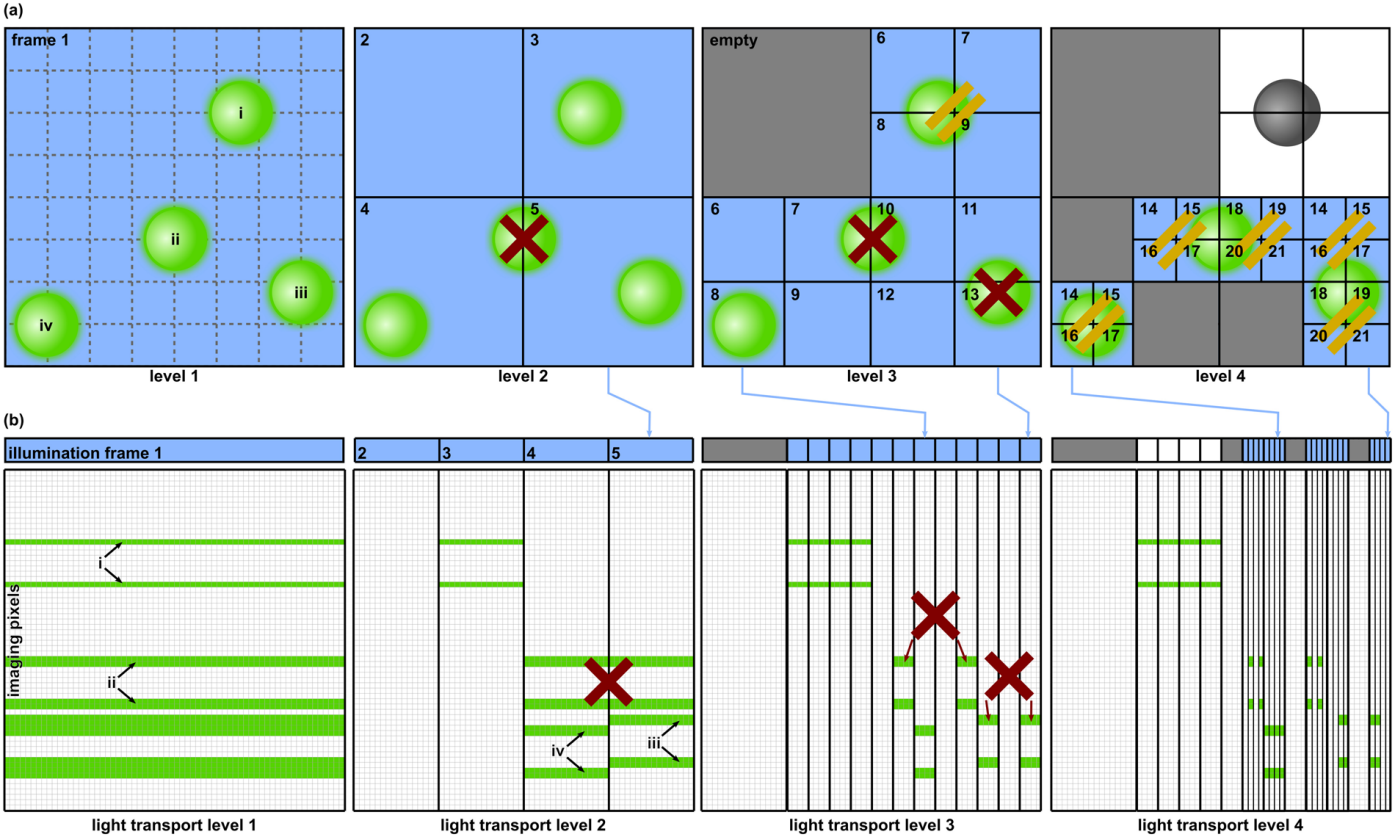
Our algorithm illustrated by a simplified 2D example with four isotropic particles (i to iv). (**a**) Top view of hierarchical illumination levels. We start by recording a frame under full illumination that is subdivided recursively. Non-conflicting frames recorded in parallel are denoted by the same frame number. Illumination rays that cause blank recordings are rejected (indicated by grey color). (**b**) Estimated versions of the light-transport matrix (64 × 64) after scanning through various levels. Conflicts are detected, and our isotropy hypothesis is applied at each level. Rows in the matrix correspond to recorded imaging rays, while columns are related to illumination rays. Emission signatures of particles (i.e., rows in the light transport) are indicated in green. Conflicts are marked by crosses, while isotropic similarities are marked by two diagonal bars. Illumination of particle i is stopped after 3 subdivision levels due to isotropic emission. Overall, the algorithm stops after scanning four hierarchical levels in the example presented.

For better illustration, we explain our algorithm with the help of the simplified 2D example in Fig. 1. Note, however, that in practice sampling is carried out in the 4D ray space’s (spatial and directional) domains of illumination and imaging light fields.

We start by recording a frame (*y*_1_) under full illumination (i.e., all illumination rays in *ϕ*_1_ are on). This leads to a first estimate of the light transport by filling all columns of *T* with *y*_1_ (reshaped as a column vector), where rows in *T* correspond to recorded imaging rays, while columns are related to illumination rays. In the next step, *ϕ*_1_ is subdivided – in our 2D example in Fig. 1 into four equal sub-illuminations *ϕ*_2_ to *ϕ*_5_. In hierarchical tree notation, the subdivided illuminations are lower-level branches of the full illumination. We then record frames with the subdivided illuminations and estimate a finer version of *T* (i.e., we update the corresponding columns in *T* with the more accurate measurements). Sub-illuminations that cause blank imaging light fields (i.e., that do not excite any particles) are rejected. Figure 1 shows such blank regions at levels 2, 3 and 4. The algorithm continues by recursively subdividing the illumination light field, recording imaging light fields, and updating the light transport matrix *T* with each new measurement. For each intermediate estimate of *T*, we determine conflicts (i.e., imaging rays that are active for multiple sub-illuminations of the same tree level). In Fig. 1, these conflicts are indicated in the light-transport matrices by rows that contain non-zero coefficients in multiple columns across different sub-illuminations. Independent (i.e., non-conflicting) sub-illuminations can be scanned in parallel. This reduces the number of scans for estimating *T* significantly. In Fig. 1, frames are recorded in parallel at the subdivision levels 3 and 4. In the best case (i.e., without conflicts), our algorithm requires 4log_4_(*m*) + 1 scans (where *m* is the total number of illumination rays), while brute-force scanning requires *m* scans. For our toy example in Fig. 1, this difference would amount to 13 to 64 scans (≈×5 speed-up/80% compression). Due to conflicts, however, 21 scans are required in our example (≈×3 speedup/67% compression). Note that the speed-up is significantly higher for realistic resolutions of the illumination light field, as we will show later. To achieve an additional speed-up, we consider the isotropic emission of the probe and avoid subdivisions down to the last (single-ray) level: We hypothesize that isotropically emitting particles cause the same imaging light-field signature (pattern) when hit by different illumination rays. Thus, we expect only a single particle to be illuminated if the imaging rays of all non-blank children are similar to the imaging rays of their parent. We make no further subdivisions in this case.

**Figure 2:**
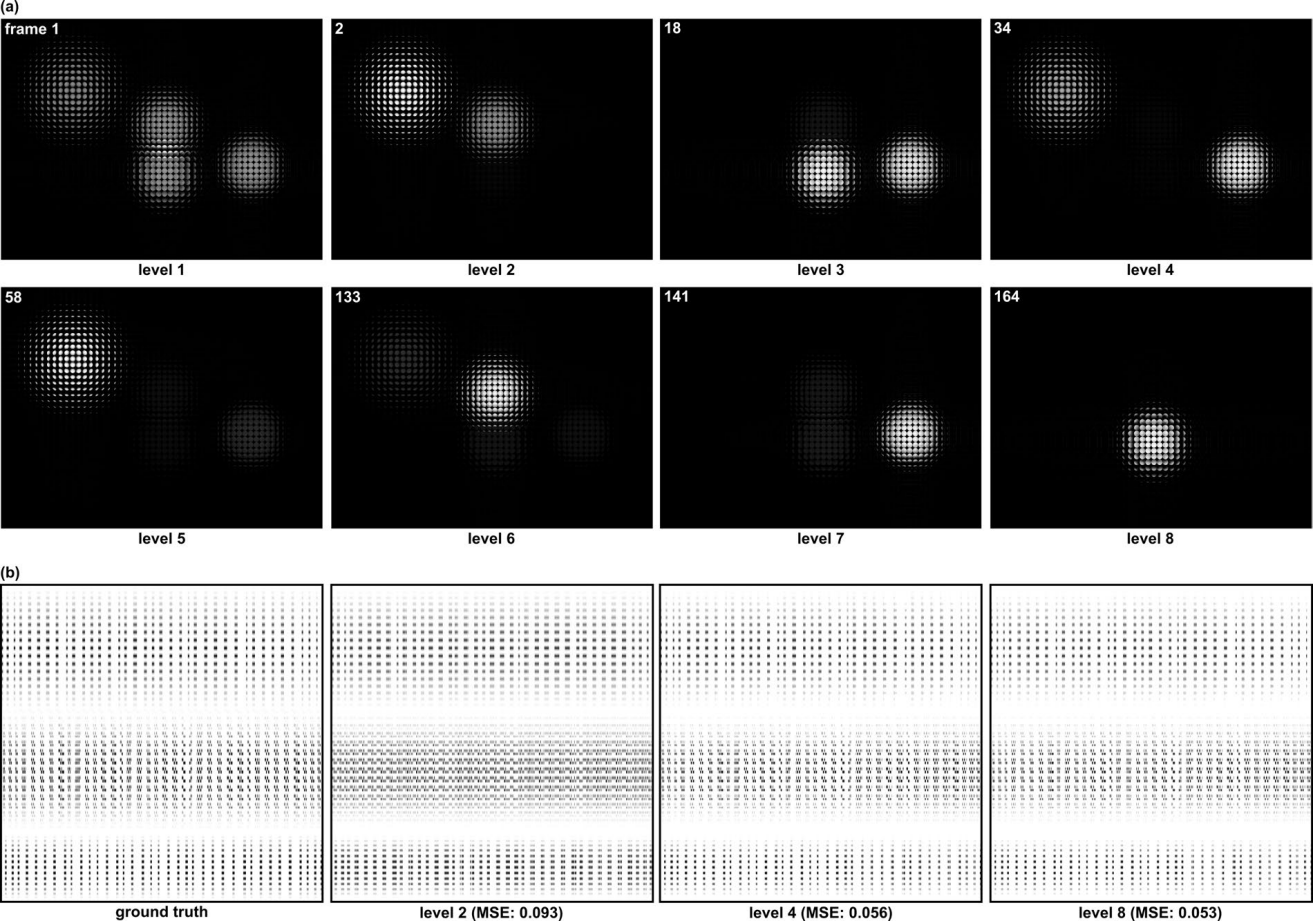
Our algorithm applied to a 4D light-field simulation of four particles. (**a**) Example imaging light-field frames (MLA recordings) for scanning levels 1 to 8. (**b**) The ground truth and updated estimates of the light-transport matrix with corresponding mean squared errors (MSE). Note that unused and empty illumination and imaging rays (i.e., empty columns and rows) are not shown in the matrix. Furthermore, we down-sampled the imaging dimension of the light transport for better visibility (from 2,881,200 to 2352 rows). The light-transport sampling processing for simulated and measured data is also shown in the supplementary video.

Figure 2 illustrates an example similar to that in Fig. 1, but with our algorithm applied to realistic 4D light-field simulations (first subdividing the spatial domain and then the directional domain). In this case, 179 frames must be scanned for 8085 illumination rays to estimate a light-transport matrix that is close to the ground truth (i.e., the result of brute-force sampling). The speed-up is ≈×45 (≈97.8% compression).

## Light-Field Factorization

We assume that the probe consists of *k* particles with isotropic emission when excited that are scanned by *l* illumination patterns as part of the light-transport sampling process. The recorded sequence of imaging light fields (*y*_1_ to *y*_*l*_) contains multiple contributions from these particles. We decompose *Y* into the independent components *Ψ* (i.e., light-field signatures of individual isotropic particles) such that

2 $$Y={\Psi }X,$$

where the coefficients *X* represent the contributions of each particle’s signature in *Y*.

While *y*_1_ (recorded at full illumination) contains contributions from all particles (resulting in dense *x*_1_), recordings under sparser illumination light fields (e.g., *y*_*l*_) will result in less excited particles and therefore in sparse coefficients. By constraining *Ψ* ≥ 0, *X* ≥ 0 (which is valid for isotropic probes), the problem in Equation 2 can be solved by non-negative matrix factorization (NMF).

While NMF is an NP-hard problem, heuristics exist that are guaranteed to converge^31^. In our implementation we apply an active-set approach to alternating non-negative least squares^32^.

## Results and Discussion

**Figure 3:**
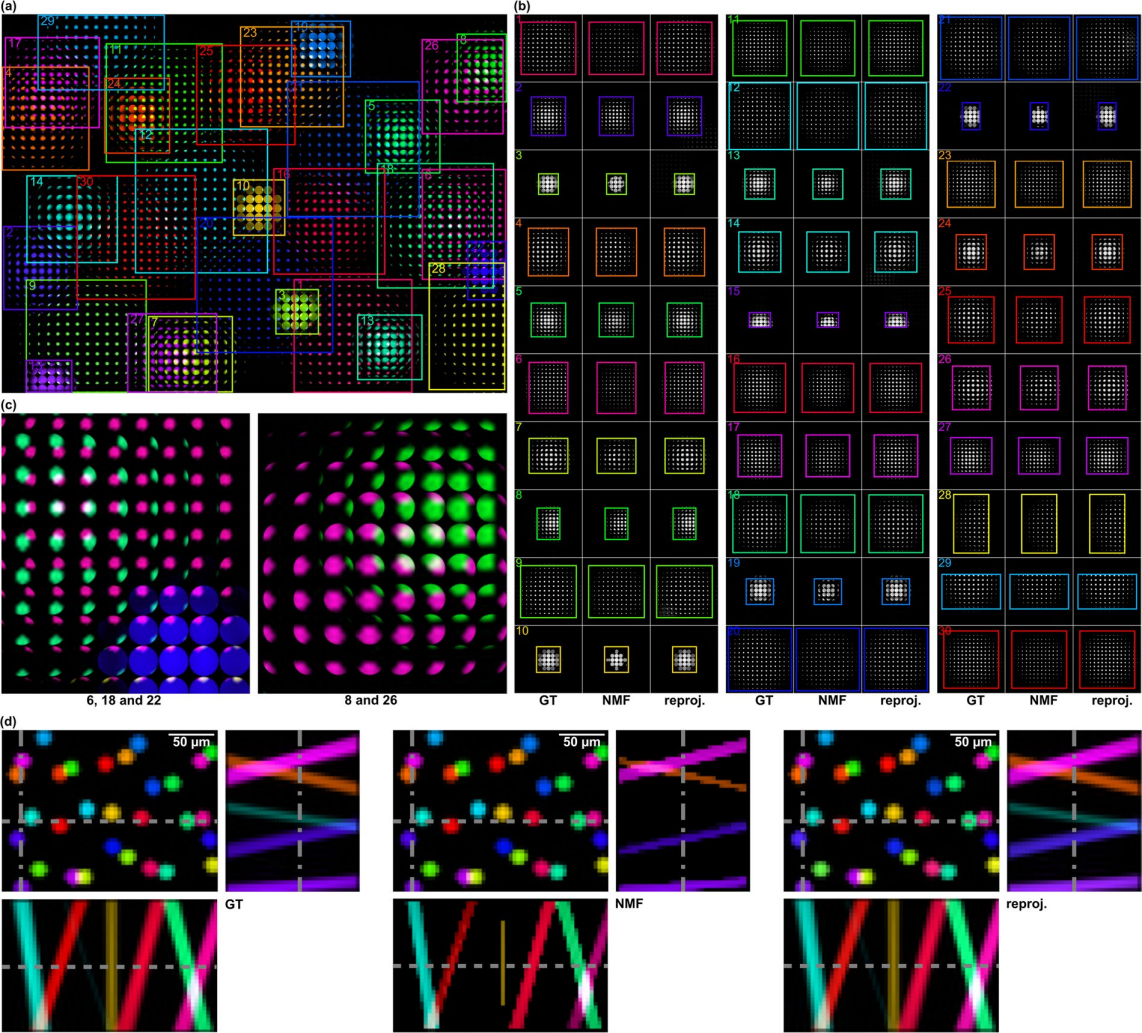
Simulated CVLE results (ground truth; after NMF; after reprojecting factorized signatures): (**a**) MLA image of color-coded particle light fields after reprojecting their factorized signatures. (**b**) Close-ups of each particle signature. (**c**) Close-ups of overlapping signatures in (**a**). (**d**) Center light-field views with corresponding space-angle (epi) slices. Space-angle representation along dashed lines in center view indicates correct handling of occlusion cases (crossings of rays). The ACD between ground truth and final results (reprojected factorized signatures) is 0.0033. Simulated results are also shown in the supplementary video.

**Figure 4:**
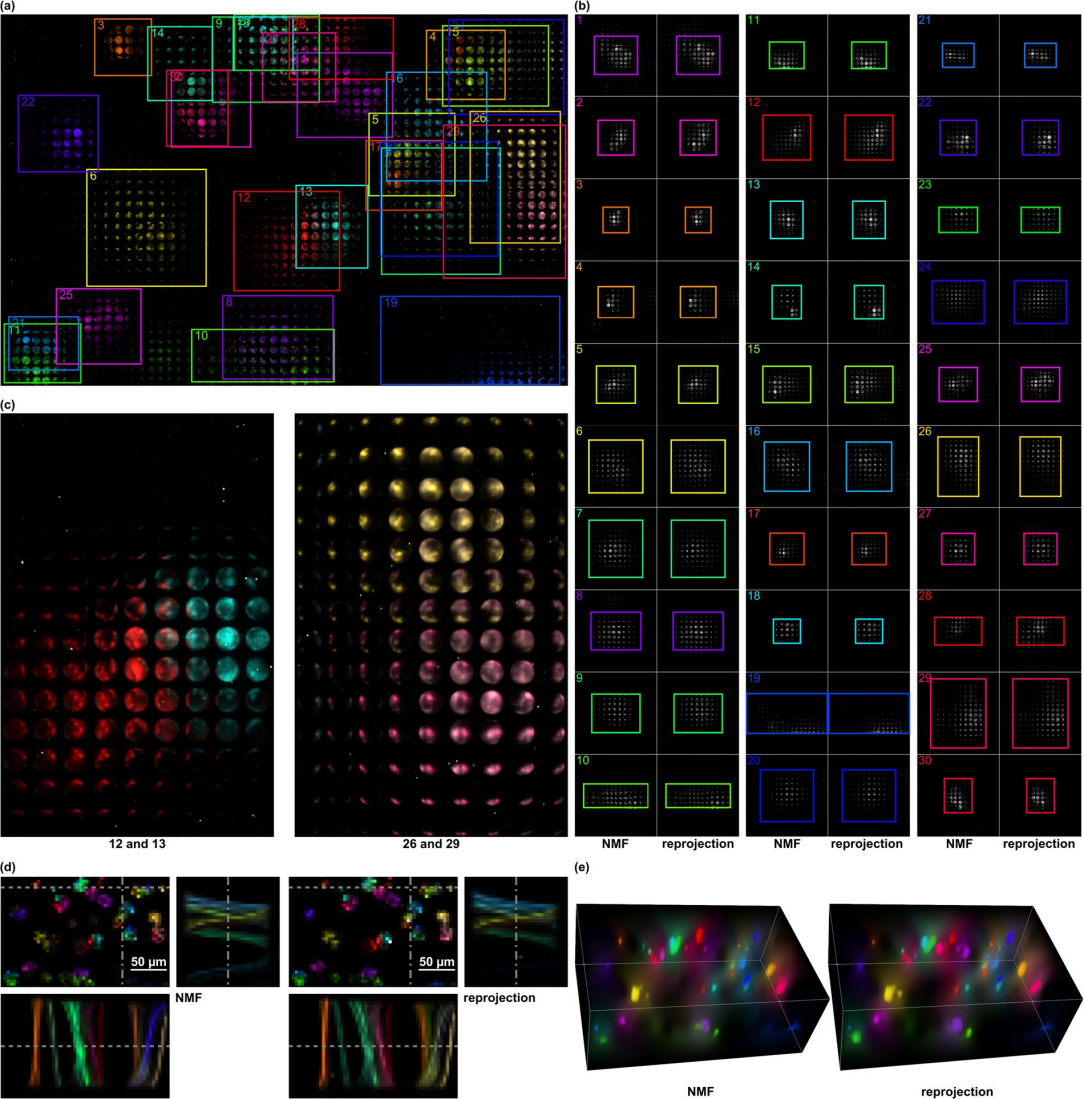
Measured CVLE results (after NMF; after reprojecting factorized signatures): (**a**) MLA image of color-coded particle light-fields after reprojecting their factorized signatures. (**b**) Close-ups of each particle signature. (**c**) Close-ups of overlapping signatures in (**a**). (**d**) Center light-field views with corresponding space-angle (epi) slices. Space-angle representation along dashed lines in center view indicates correct handling of occlusion cases (crossings of rays). (**e**) Volumetric renderings (perspective projection along axial direction of center view). The ACD between NMF and final results (reprojected factorized signatures) is 0.1177. Note, that a ground truth does not exist in this case. Measured results are also shown in the supplementary video.

Figure 3 presents a light-field simulation of a more complex probe (dimensions: 100 μm axial, 175 μm × 234 μm lateral) with 30 particles (20 μm diameter fluorescent microbeads). The simulated light-field segments had (spatial × directional) resolutions of 42 × 56 × 35 × 35 (imaging) and 11 × 15 × 7 × 7 (illumination). A total of 845 scans was required by our algorithm to estimate the light-transport matrix (speed-up of ≈×10/90% compression compared to brute-force scanning). Since a ground truth exists for the simulation, we can determine an average cosine distance (ACD) error of 0.0033 when comparing the ground truth light-field signatures with the light-field signatures that result from reprojecting the factorized signatures (i.e., from exciting single particles only). The latter is achieved by projecting the light-field illumination *ϕ*_*i*_ computed from *y*_i_ = *Tϕ*_*i*_, where *y*_*i*_ is the factorized light-field signature of a single particle. Figure 4 illustrates measurement results acquired in experiments using the prototype described in the methods section and under similar conditions as in the simulations shown above: around 30 (10 μm to 20 μm) fluorescent microbeads embedded in a polydimethylsiloxane carrier, scanned in a section of 100 μm axial, 150 μm × 230 μm lateral, with light-field resolutions (spatial × directional) of 36 × 55 × 35 × 35 (imaging) and 11 × 15 × 5 × 5 (illumination). In total, our algorithm required 561 scans to estimate the light-transport matrix (speed-up ×7.4/86% compression). Since a ground truth does not exist in this case, we can determine an ACD of 0.1177 only when comparing the factorized light-field signatures with the light-field signatures that result from reprojecting the factorized signatures (i.e., from exciting single particles only). Note that in contrast to the simulation, this can only indicate the quality of the light-transport matrix, but ignores the error introduced by the factorization.

The results presented above reveal that, up to a remaining precision error arising during light-transport sampling and light-field factorization, our approach supports simultaneous, controlled concentration of light at multiple volumetric positions by means of a 4D illumination light field. It avoids 3D reconstruction of the probe, deconvolution, and calibration of optical elements, while speeding up light-transport sampling by up to one order of magnitude (85–90% compression).

Since scattering is included in the light-transport matrix, it is taken into account when solving *y*_*i*_ = *Tϕ*_*i*_ for *ϕ*_*i*_. Given that the factorized probe signatures *y*_*i*_ can be determined, scattering during the illumination is considered in *ϕ*_*i*_.

**Figure 5:**
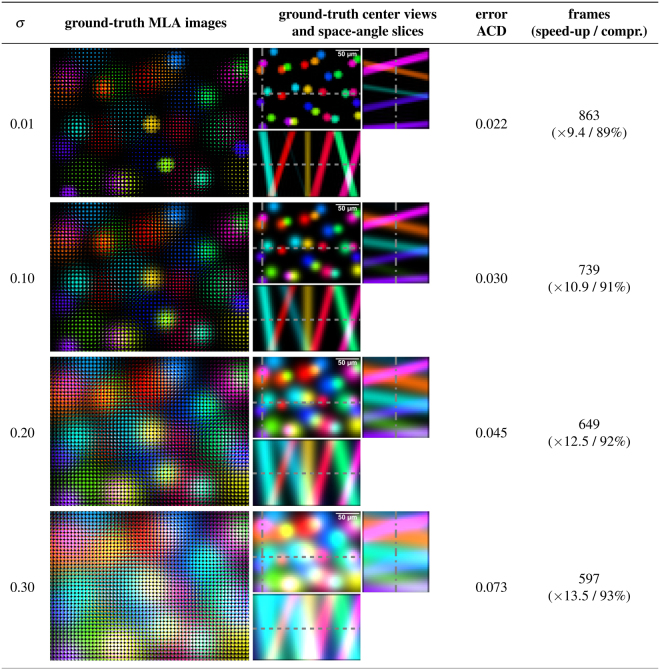
Simulated CVLE results with different amounts of scattering (*σ*). With increasing scattering, the signatures of individual particles become more isotropic, as shown in the MLA images and the center views with corresponding space-angle slices. The ACD between ground truth and reprojection represents only the error in the light-transport estimation, as we assume a perfect light-field factorization in this example. Results for simulated scattering are also shown in the supplementary video.

Figure 5 presents simulation results for a probe with increasing amount of scattering. We model scattering as homogeneous and Gaussian-distributed in angle^29^, where a higher scatter coefficient *σ* indicates more scattering. Additionally, we assume perfect factorization results. With increasing scattering, the reprojection error rises and the number of frames required for measuring the light-transport matrix decreases. The latter is the result of more isotropy from scattering in the light transport that causes our scanning to stop early.

Our approach has several limitations. First, NMF requires a manual estimate of the number of particles present in the probe. Evaluating more advanced factorization techniques, such as model-driven approaches^13,29^, that support automatic estimation and provide better factorization results will be part of our future work. Currently, our approach (like other factorization-based techniques^13,28^) is limited to spatially static (i.e., fixed) probes. A dynamic update of *T* for tracked particles of a moving probe and experiments with living organisms will be investigated in the future. Furthermore, our hierarchical light-transport sampling results in low amounts of light at finer illumination levels, and thus leads to low signal-to-noise ratios or long camera exposure times. At present, we increase the exposure time with finer sampling levels. More advanced illumination strategies (e.g., similar to Hadamard patterns) and high-dynamic range camera systems will help to overcome this issue. The limited spatial and directional resolutions of our prototype can be overcome by applying higher resolution SLMs, imaging sensors, and MLAs.

## Method

**Figure 6:**
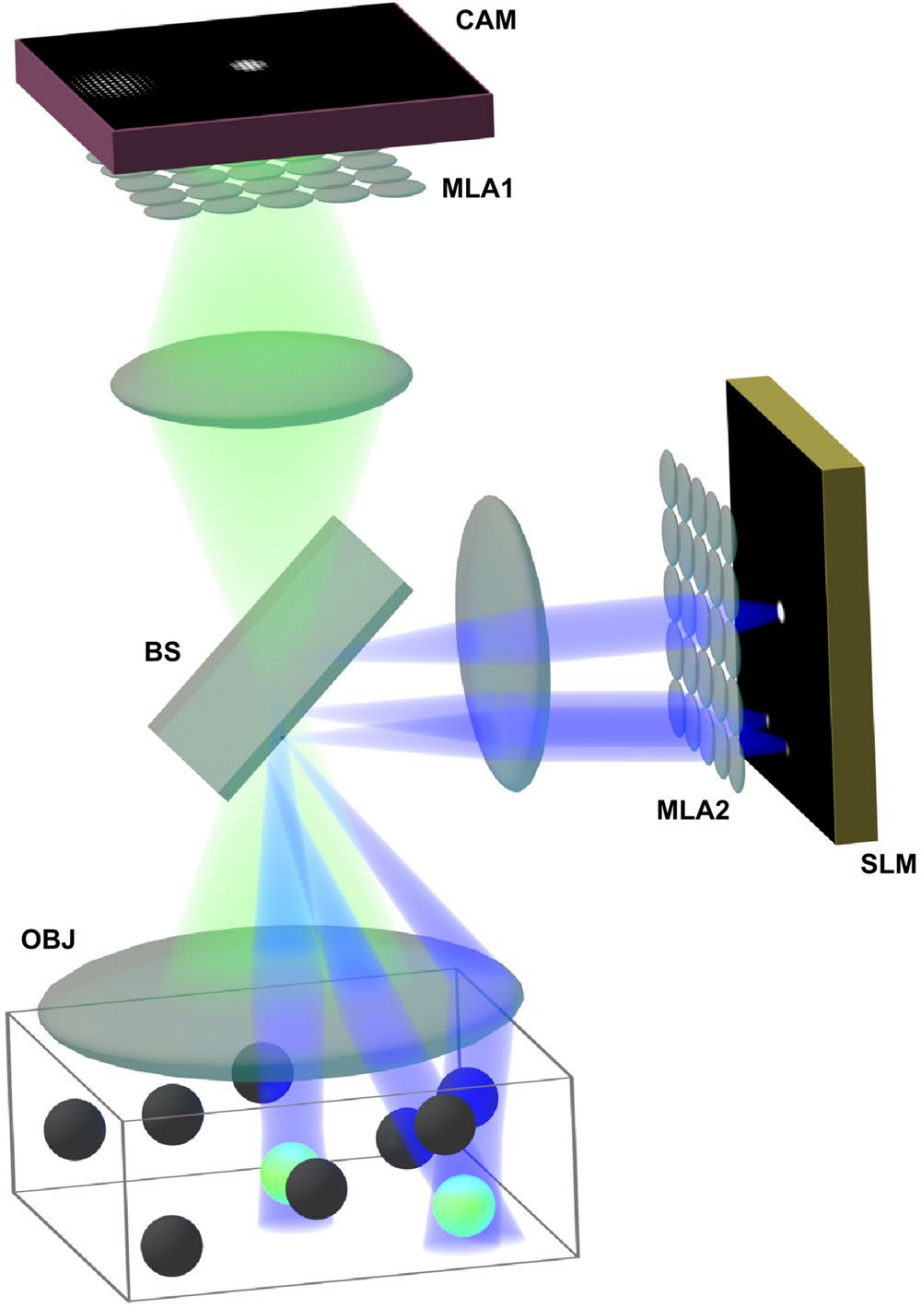
Optical layout of our light-field microscopy prototype. The 4D illumination pattern is generated by a spatial light modulator (SLM) and concentrated on the probe by a microlens array (MLA2) and the objective (OBJ). Emissive light from the probe is propagated on a camera sensor (CAM) via the objective (OBJ) and another microlens array (MLA1). The beam splitter (BS) and corresponding filters restrict the light in the optical paths to certain wavelengths. Example illumination and imaging light fields are shown on SLM and CAM.

In our experiments we used a light-field microscope prototype^12^ as shown in Fig. 6 and explained in more detail by Schedl and Bimber^15^. We equipped a 60×/1.2NA objective with a 250 μm imaging (MLA1) and 300 μm illumination MLA (MLA2) of focal lengths 6250 μm and 7500 μm, respectively. We used a Texas Instruments Digital Light Processor at a resolution of 340 × 256 (pixel size 41.1 μm) as spatial light modulator (SLM). Note that we used the SLM at reduced resolution when compared to Schedl and Bimber^15^. The light source was an 120 μm metal halide lamp (X-Cite 120 μm, EXFO) and we placed the corresponding filters in a standard Nikon microscopic filter cube. For recording (CAM) we used a Retiga 4000 R monochrome camera with 2048 × 2048 resolution (pixel size 7.4 μm). In front of the imaging and illumination MLAs we placed 1:1 relay lenses; two Nikon AF Nikkor with focal length 50 mm mounted nose-to-nose and a telecentric relay lens from Brilliant Technologies, respectively.

The fluorescent microbeads used in the experiment shown in Fig. 4 were 10 μm to 20 μm Green Polyethylene microspheres (material density 0:99 to 1:01 g cm^−3^; peak excitation 470 nm; peak emission 505 nm; distributor cospheric). The polydimethylsiloxane carrier in which they were embedded was Elastosil RT 604 from Wacker (material density 0.97 g cm^−3^).

For solving Eqn. 2 (NMF) we used the Matlab implementation of the non-negative least-squares active-set algorithm from^32^ with 200 iterations. When reprojecting individual signatures (solving for *ϕ*_*i*_ in *y*_*i*_ = *Tϕ*_*i*_) we used 500 iterations of the simultaneous algebraic reconstruction technique (SART)^33^ and binarized the 4D illumination pattern. The source code for our simulated CVLE experiments (i.e., Figs 3 and 5) is available at https://github.com/JKU-ICG/CVLFE.

## Electronic supplementary material

Supplementary Video

Supplementary Video Legends
