# Supplementary material for: Compressive Volumetric Light-Field Excitation
Source: Sci Rep. 2017 Oct 25;7:13981. doi: 10.1038/s41598-017-13136-2 (PMC5656577; doi:10.1038/s41598-017-13136-2)
Supplement: Supplementary file 2 — Supplementary Video Legends [file 41598_2017_13136_MOESM2_ESM.pdf]

Supplementary Information: Compressive Volumetric Light-Field Excitation

David C. Schedl[1] and Oliver Bimber[1,*]

[1]Faculty of Engineering and Natural Sciences, Johannes Kepler University, Linz, 4040, Austria
*oliver.bimber@jku.at

Supplementary Video Legends

Supplementary Video 1: Following sequences are shown:

- Imaging light-field frames (MLA recordings) with updated light-transport matrix ($\boldsymbol{T}$) during light-transport sampling for simulated and measured CVLE results. For illustrative reasons we down-sampled the imaging dimension of the light-transport matrices. Our algorithm recorded 845 and 561 frames for the simulated and measured data, respectively.
- Space-angle (epi) representations of simulated and measured CVLE reprojection results. Each particle reprojection is differently colored and the spatial location of the space-angle slices is animated.
- Volumetric renderings (perspective projection along axial direction of center view) of single particle and multiple particles exposures. Corresponding MLA images are shown on the right side.
- Space-angle (epi) representations of simulated CVLE reprojection results at varying scattering levels (σ). The results only showcase the light-transport estimation, as we assume a perfect light-field factorization in this example.
- Imaging light-field frames (MLA recordings) showing single and multiple particle exposures of simulated CVLE results with different amounts of scattering (σ). With increasing scattering the signatures of individual particles increase in size.
